# On the State of Medicine in Norway

**Published:** 1837-10

**Authors:** Frederick Holst

**Affiliations:** Professor of Medicine at the Royal Frederick's University in Christiania


### PART FIFTH.

### Medical Intelligence.

---

### ON THE STATE OF MEDICINE IN NORWAY.

By Frederick Holst, M.D., Professor of Medicine at the Royal Frederick's University in Christiania.*

The state of the sciences in Norway is but little known out of Scandinavia, the native works being but few, and, generally speaking, inaccessible to the majority of the cultivated nations of Europe, on account of the language in which they are composed. Moreover, we are seldom visited by scientific travellers; and the few that come among us are not only deficient in the requisite knowledge of our language, without which it is impossible for them to derive much advantage for their visit, but also generally remain for such a short period that they depart with very superficial, and not unfrequently even incorrect, notions of the country, its inhabitants, and its institutions. These general observations are peculiarly applicable to medical science. It is consequently no easy task to prepare a Report on the State of Medicine in Norway, which shall at the same time be sufficiently concise to be suited for a Journal, and sufficiently full to give foreign readers a tolerably clear idea of the subject.

### THE UNIVERSITY.

The want of a university in Norway had long been felt, when his Majesty King Frederick VI., by his decree of the 2d September, 1811, founded that in Christiania, by the name of *Universitas Regia Fredericiana.* After the necessary preparations, this institution was opened in the summer of 1813: in the ensuing year, Norway was separated from Denmark, and the illustrious founder was thereby prevented from fully completing its organization. Its regulations were at first the same as those of the university of Copenhagen, except so far as local circumstances made alterations necessary; but, on the 28th of June, 1824, it received its own charter, which, along

* It may be necessary to observe that, as the language used in Norway in writing, and by all educated persons in speaking, is, though called Norwegian (*Norsk*), identically the same as that written and spoken in Denmark,—i. e. as the Danish,—we have, to prevent confusion, always used the latter appellation, as more generally known. The language spoken by the peasantry of Norway differs from the last by the retention of many words of the ancient Scandinavian, which are no longer preserved in the Danish or else have undergone greater modifications: as may be supposed, the dialect spoken in the mountain districts approaches the nearest to the old Scandinavian; from which latter the present Icelandic differs but little, the remote situation of the island and other causes having preserved the language nearly in the same state as when the original colonists brought it with them from Scandinavia. We also wish it to be understood that, as there is no single word in English which answers to the Danish *Læge,* or medical practitioner, it has been sometimes rendered physician and sometimes surgeon: in Norway, as will be seen by the Report, there is no distinction of the two classes, as amongst us; the functions of both being always understood to be combined. In the titles of persons filling official situations, however, a distinction is sometimes made by the use of the terms *physicus* and *chirurg* in composition, as in the words *Stadsphysicus, general-chirurg;* but the holders of these situations are still equally styled *Læger.* The vernacular term for surgeon is *Saarlæge* or *Vundlæge,* just as the Germans use *Wundarzt,*—i. e. wound-physician or wound-doctor. *Læge* corresponds to the Anglo-Saxon *læc* or *læce,* whence the antiquated English *leech.*—Translator.

with sundry special regulations, treats of its constitution, privileges, and organization; of its academic prælections, festival days and vacations, examinations, discipline, exhibitions, and degrees; of the use of the library and other scientific collections; and of its income and expenditure.

The officers of the university are, a chancellor, a vice-chancellor, nineteen professors, six lecturers, and one tutor, (divided into the four faculties of theology, jurisprudence, medicine, and philosophy,) a librarian, a sub-librarian, a treasurer, a secretary, and a curator of the botanic garden, who are all named by the king. It is represented and governed by the Academic College, which consists of the vice-chancellor, a dean of each faculty, and two assessors of the faculty of philosophy; seven in all. The vice-chancellor is, by the charter, a permanent member of the College; the rest sit and vote only for the year, and are then replaced by others.

Matters which the members of the university are not empowered to decide upon themselves are referred to the chancellor, who reports through the proper office whatever is to be laid before the king.

Each professor is bound to deliver the public lectures required by the nature of his office, without any fees from his auditors; but he has likewise liberty to give private lectures in any department whatever for a stipulated honorarium: these private lectures, however, have as yet been delivered only by a few, and that not constantly. The student may consequently pass through his whole academic course without having to pay a single fee. Examinations, graduations, and diplomas are likewise free of charge. The year is divided into half-years; the first lasting from the 1st of January to the 30th of June, and the second from the 1st of July to the 31st of December. The lectures are held during the whole year, except in the winter and summer vacations, (the former being from the 16th of December to the 15th of January, and the latter from the 16th of June to the 31st of July,) and on holidays and the university festival days; viz. the anniversary of the reformation, the king's birthday, and commencements, all of which are solemnized by a Latin oration by one of the professors; they are also discontinued during the holding of examinations, which, on account of the annually increasing number of the students, occupy a considerable time. Thus, in the faculty of medicine, six or seven months of each year are taken up with examinations and the vacations, and there are consequently but five or six left for lectures.

Whoever wishes to be admitted as a *civis academicus,* or student of the university, must undergo an *Examen artium,* at which he has to display his proficiency in Danish, Latin, Greek, French, German, divinity, history, geography, geometry, and arithmetic; and, if a divinity student, in Hebrew also. When he has passed this examination, he is enrolled as a *civis academicus,* engages to keep the academic laws, and chooses one of the professors for a tutor, *(privat præceptor,)* who is to be his especial adviser and guide both in literary and scientific and other matters, and to keep watch over his conduct while at the university. The student has next to pass an *Examen philologico-philosophicum,* at which he is examined in Greek and Roman literature, history, stereometry, trigonometry, the elementary principles of algebra, physics, astronomy, natural history, logic, psychology, metaphysics, and ethics. These two examinations are held by the faculty of philosophy, and are the same for all the students, be their intended professional course what it may.

Medical Instruction. The student who, after standing the above-mentioned examinations, intends to devote himself to medicine, now passes over to the faculty of that science, whose dean gives him a synopsis, *(Studieplan,)* which indicates the order he has to follow in studying its various departments, and whose five professors, in conjunction with those of natural history and chemistry, from the faculty of philosophy, deliver lectures on its several branches, both theoretical and practical. The order prescribed in the medical *Studieplan* is as follows:—The student is first to apply himself to natural history and chemistry, as being the true foundation of the healing art. He is next to learn anatomy, both from lectures and by dissecting, and is expected at the same time to attend to comparative anatomy, for which he has been the more fitted by his previous studies in zoology. Physiology may also be studied at this period. The student is especially recommended to commence early to attend an hospital, for the purpose of satisfying himself whethe he has really a taste for the medical profession. The fundamental branches of knowledge being thus acquired, the student, without laying them aside, is now to proceed to their application in the departments of pharmacology (general and special), pathology and therapeutics (both medical and surgical), and midwifery; and is to conclude with medical jurisprudence, and the literary history of medicine, as well ancient as modern. The plan, of which this is the outline, was drawn up, in 1826, by Professors Thulstrup, Skjelderup, Sörenssen, and Holst.

Instruction in the practice of medicine and surgery was formerly given in the City Infirmary, but, since the autumn of 1826, it has been given in the National Hospital, which was then opened. This institution has been founded and supported almost entirely at the public expense, and, when completed, will be capable of accommodating two hundred ordinary patients, seventy-five syphilitic or affected with cutaneous diseases, and twenty-five lying-in women; consequently, three hundred patients in all. It receives about one thousand annually. The university professors of the practice of medicine and of surgery are its head medical officers, and give daily clinical instruction there. There are, besides, three physicians extraordinary, who have to pass an examination for their office; four candidates, who may at the same time be students; and an indefinite number of volunteers.

With these aids, the student prepares himself for his medical examination, which is conducted as follows:—1st. He is required to perform an anatomical dissection of a given portion of the human subject. 2d. A medical question is propounded, to which he has to give a written answer.* 3. He is given a medical and a surgical case to examine in the National Hospital, and is required to give in writing their history, diagnosis, prognosis, and treatment. 4. He undergoes an oral examination, which lasts three days, and occupies altogether about nine or ten hours, during which he is examined in natural history, chemistry, anatomy, physiology, pharmacology, toxicology, medical and surgical pathology and therapeutics, midwifery, and forensic medicine. These examinations are held twice a year, and seven days are devoted to each candidate. The first three trials are conducted solely by the medical faculty; the fourth by the same in conjunction with the professors of natural history and chemistry from the faculty of philosophy. At the completion of the examinations, the candidate receives, according to his deserts, as determined by certain rules, one of the following titles: *Laudabilis, Haud illaudabilis, Non contemnendus.* He then gets a testimonium as *Candidatus Medicinæ*, and as such is entitled both to practise and to become a candidate for public medical situations.

As Norway, after its separation from Denmark, wanted candidates to fill the many vacant medical and judicial situations, and there was no chance of there being soon a sufficient number of regularly matriculated students to supply the deficiency, it was deemed advisable to facilitate the access to the juridical and medical qualificatory examination. Accordingly, by a law of the 6th of June, 1816, a so-called *preliminary examination* was authorized, which was limited to Danish, a little Latin, arithmetic, history, geography, and divinity. Any one who passes this may, without farther trial, prepare himself at the university, and undergo the medical qualificatory examination. This preliminary examination is therefore a substitute, to those who avail themselves of it, for both the *Examen artium* and the *Examen philologico-philosophicum* of the regular *Civis academicus;* but it must be evident to any one, on comparison, that a Preliminarist cannot be nearly so well prepared to begin his medical studies as a Candidatus philosophiæ; i.e. one who has undergone the two last-mentioned examinations. Accordingly, the faculty of medicine, in a report of the 23d of August, 1832, represented to his majesty's government in Norway, that the acquirements necessary for the preliminary examination could not be considered sufficient to enable the medical student to acquire a sound knowledge of his profession;

* The following were the questions proposed in the present year: at the spring examination, "Explicare et dejudicare præcipuas de medicaminum actione theorias;" at the autumn, "Quibus signis tumor scroti qui hydrocele tunicæ vaginalis testiculi dicitur, ab aliis scroti tumoribus dignoscitur?"

that the number of regularly educated students was then, and was likely to continue, sufficiently great to supply the kingdom with the requisite number of qualified practitioners; and that, therefore, the state of affairs which had required the law of the 6th of June, 1816, had nearly or altogether ceased to exist. In conformity with this report, his majesty, in the year 1833, had a proposition laid before the *Odelsthing*,* that the law of the 6th June, 1816, should be repealed, so that no one should thenceforth be permitted to study medicine at the university without having passed the *Examen artium* and *Examen philologico-philosophicum.* But this proposal, after long and warm debates, was rejected by a considerable majority, although the measure had been recommended by all the competent authorities that had considered and reported upon it.

Accordingly, there are still two kinds of medical students at the university,—*Cives academici*, or those that have been matriculated, and *Preliminarists*, or those who have passed only the examination whence they take their name. The medical examination of the latter embraces the same subjects and is conducted in the same manner as that of the former, with the exception that, both in writing and speaking, the language used is Danish, and not Latin, and that the titles are as follows: *First rate; second rate; third rate.* Whoever passes this examination receives a *testimonium* as *Examinatus medicinæ*, and is entitled to practise medicine, but not to obtain medical situations for which a higher grade of education is required; such as professorships and tutorships in the university, and the posts of surgeon or physician general in the army.

The medical examination, as conducted at the university of Christiania, justly deserves the name of *Examen rigorosum.* From May, 1817, when it was first held, to the present date, there have passed fifty-four who had matriculated, (twenty-five with Laudabilis, twenty-seven with Haud illaudabilis, and two with Non contemnendus,) and sixty-nine Preliminarists, (ten with first rate, fifty-three with second rate, and six with third rate.) In general it requires five years' preparation, a very few have passed it after four years, and the majority require more than five.

As to degrees, there are two in each faculty, namely, that of licentiate and that of doctor. The Candidatus medicinæ, who wishes to take these degrees, must, in the first place, have passed both the philologico-philosophical and the medical examination with the character of Laudabilis. He has next to write a thesis, in Latin, on some medical subject of his own selection, and, on its being accepted by the faculty, he has to hold a *learned colloquium*, in the presence of the Academic College, with the appropriate professors in the faculty of philosophy, on Greek and Latin literature, and on history and philosophy. This examination also being found satisfactory, the Academic College requests permission of the king, as well for the candidate to defend his thesis publicly, as for the university to confer on him the degree of Licentiate, in case his defence shall be considered deserving of it. The royal permission being obtained, the candidate publicly defends his thesis in the theatre of the university, in presence of the faculty, and, on their approval of the manner in which he has performed this exercise, the degree of licentiate is conferred on him.

If he wishes further to obtain the degree of doctor, he must, in presence of the faculty and the Academic College, deliver three public prælections on subjects of his own choosing, (previously, however, announcing them to the Academic College,) and also compose an inaugural dissertation in Latin on some medical subject, likewise of his own selection. The dissertation being accepted by the faculty, the candidate delivers the prælections, and afterwards publicly defends his dissertation, (both acts by special royal permission;) and, these trials also being satisfactorily passed, he is publicly proclaimed *Doctor medicinæ*, and presented with a diploma as such. These exercises, as hitherto performed, have occupied from six to eleven hours. The gra-

* In Norway, laws are passed by the king and the *Storthing*, or parliament, in conjunction. The Storthing consists of two chambers, the *Odelsthing* and the *Lagthing*: every proposed law is laid before the former, and, if approved of, sent up to the latter; if both agree, the resolution is reported to the king, on whose sanction it becomes a law.

duation is generally performed, on the part of the university, by the dean of the faculty; but any other of its members the candidate may wish is competent to act. The ancient ceremonies of the open and shut book, the cap, and the ring, are still used; but the kiss and the oath are omitted.

Every one who has obtained a doctor's degree in the university of Christiania is entitled to hold lectures there; but, as this is the only privilege it confers,—as academic degrees are not necessary to enable physicians to hold office,—as those who have passed their medical examination usually get some public or private employment out of the city, and but few have an opportunity of continuing at the university for several years after that period, there have been but three such degrees taken during the twenty-three and a half years that have elapsed since the opening of the university; namely, one in 1817, and two in 1830. In the other faculties there have been no exercises for doctor's degree whatever performed.

Dispensations from the regulations above described may occasionally be obtained: for instance, foreigners may be matriculated without undergoing the ***Examen artium***, on proving their matriculation at some other university, and producing a proper ***testimonium vitæ***. Likewise, persons of acknowledged learning and literary merits, who have graduated at another university, or have acquired a high character by their writings, may be exempted from one or more of the above trials, and yet receive degrees.

The number of students is constantly increasing in all the faculties. At the close of last year they amounted to 724; and, with the addition of those matriculated in the present year, the entire number of students now at the university amounts to about 800; a much greater than is required to supply the kingdom with officers. The medical students have become more numerous in proportion than any other: there were forty in 1827, and there are now fully 120.

The university possesses the following establishments and collections:

1. A Library, founded by donations from King Frederick VI. and various private individuals, and, since that, yearly augmented by purchases; so that it now contains about 120,000 volumes. Between eighteen and nineteen thousand volumes are lent out in the year.

2. The Botanic Garden, established at Toïen, an estate situated about an English mile from the city, and presented by King Frederick VI. It contains hothouses, a residence for the curator, a lecture-room, upwards of 6000 plants, and a considerable e rbarium of dried specimens.

3. A Museum of Natural History, containing several thousand stuffed animals and minerals.

4. A collection of physical and chemical instruments.

5. An Observatory, and a collection of astronomical instruments.

6. A collection of Coins, which, at the end of the year 1835, contained 14,596 specimens.

7. A collection of Records, to the amount of 6,200.

8. A collection of Models, 168 in number.

9. A collection of Northern Antiquities, 712 in number.

10. An Anatomical Museum, the groundwork of which was laid by a donation of Professor Skjelderup's, and which contains 966 specimens.

11. A collection of surgical and obstetrical instruments, bandages, and machines; 548 in all.

12. A pharmacological collection of 648 specimens.

N.B. The amounts mentioned in the six last numbers are those ascertained at the end of the year 1835.

The library is open two hours for the first five week-days in each week; the other collections on particular days every week.

The disbursements of the university amount to about 50,000 specie-dollars, (*Spd.*) or, at the present course of exchange, 10,000*l.* sterling, yearly. The particulars of the expenditure are as follow:

| | |
|---|---|
| Salaries and wages to the officers and assistants, about | 37,000 *Spd.* |
| Stipends, pensions, and exhibitions . . . | 1,700 |

| | |
|---|---|
| The scientific collections . . . . . . | 5,500 *Spd.* |
| The botanic garden . . . . . . | 1,000 |
| Tour through the country, to study its natural history | 400 |
| Expenses of the establishment . . . . . | 4,400 |

Its income consists of the interest of its funds, tithes, rents, profits on the sale of calendars, registry fees from the students, and one-third of the revenues of the board of education; altogether amounting to about 17,000 *Spd.*; together with a yearly contribution from the public purse of from 30,000 to 33,000 *Spd.*

With respect to their salaries, the professors are divided into four classes, according to their standing; the corresponding salaries being 1,050, 1,200, 1,350, and 1,800 *Spd.* yearly. The lecturers have 750 *Spd.*

| | |
|---|---|
| The library receives an annual grant of | 3,000 *Spd.* |
| The anatomical museum gets at present, | 470 |
| The collection of surgical instruments, | 180 |

and so forth.

The following professors constitute the present *Faculty of Medicine:*

1. Dr. Michael Skjelderup, Anatomy, Physiology, Forensic Medicine.
2. Dr. N. B. Sörenssen, Medical Pathology, Therapeutics, and Clinique.
3. Dr. M. A. Thulstrup, Surgical Pathology and Therapeutics, and Midwifery.
4. Dr. Frederick Holst, Pharmacology, Toxicology, *Politia Medica.*
5. Dr. Christian Heiberg, Anatomy, Surgery, Clinical Surgery.

The following professors belong to the *Faculty of Philosophy,* but are attended by medical students:

1. J. Rathke, Natural History.
2. J. Keyser, Physics, Chemistry.
3. J. Esmark, Mineralogy.
4. B. M. Keilhau, Mineralogy.
5. M. N. Blytt, Botany, (lecturer.)

APOTHECARIES.

When any one wishes to devote himself to the study of pharmacy, with a view of becoming qualified to apply for a royal licence to keep an apothecary's shop in Norway, he serves as apprentice for a certain number of years with a licensed apothecary, and, on the expiration of that period, after undergoing an examination by the physician of the town or district, becomes an assistant. He must then, according to a royal ordinance of the 4th December, 1672, pass the pharmaceutical examination, which, as long as Norway was united with Denmark, was held at Copenhagen for both nations in common, but, since their separation, takes place in Christiania. This examination used to be merely oral; but now, by a law of the 2d June, 1836, written and practical exercises are also required. The candidate is first required to deliver in a written dissertation on a proposed pharmaceutical subject; he is then orally examined in natural history, chemistry, pharmacognosis, the reading of prescriptions, pharmacy, the estimate of the commercial value and the qualities of drugs, and the valuation of prescriptions; lastly, he has to analyze two pharmaceutico-chemical substances, and to make a medical preparation, giving in, at the same time, a written account of the process he employs. The examination is held twice yearly by a board, consisting of the university professors of natural history, chemistry, and pharmacy, together with one of the apothecaries of Christiania. From 1816, when it began to be held in Norway, up to the present time, there have been thirty-four candidates examined.

An important step towards the more successful and better-grounded study of pharmacy in Norway has certainly been made by the regulation of the 2d of June, 1836, just mentioned; but it still remains to provide that no one shall be taken as apprentice without being possessed of a sufficient general education; and it is to be hoped that this defect will be remedied by the forth-coming medical regulations.

### MIDWIVES.

As long as Norway was united with Denmark, the females who wished to become midwives had to go to Copenhagen, to receive the necessary instruction at the obstetric school instituted there for the purpose, and to pass the requisite examination, without which they could not receive their appointment as midwives. The separation of the kingdoms rendered necessary the erection of a similar school in Norway, in which lectures on the art were commenced in 1815, while, in 1818, an institution was opened for lying-in women, capable of accommodating fourteen at a time, and destined for the instruction of medical students and midwives in the management of females during and after parturition, and of new-born infants; into which from 100 to 150 women are received annually. The master of the school, who is at the same time chief physician to the institution, gives the requisite instruction: this place has been heretofore filled by the university professor of obstetrics. The course lasts a year, and examinations are held yearly by the master, in conjunction with the members of the faculty of medicine. No fees whatever are required from the students, as the Storthing grants from the public purse a sum sufficient to defray the requisite expenses, (of late years, 1,650 *Spd.* yearly:) many of the pupils are sent at the expense of their communes, and are consequently even supported during their stay. The examination, from the time it first commenced, in 1816, up to the present period, has been passed by 320 women, making on an average fifteen a year; while, during the union with Denmark, only about four were sent to Copenhagen yearly.

### VACCINATION.

The practice of vaccination is enjoined in Norway by a law of the 10th April, 1810, by which inoculation for small-pox is prohibited under a penalty; and no one can attend a public school or institution, be taken as apprentice, confirmed, married, or enlisted, without proof of having previously been successfully vaccinated, or else of having had small-pox. The operation is performed by every medical practitioner who has passed his examination, and by persons of both sexes who, though not practitioners, have been taught how to vaccinate, and recommended by physicians for licence as assistant vaccinators. The person vaccinated is not required to pay any fee, but the operator is entitled to a small honorarium from the public purse for each successful case. There is reason to believe that vaccination is more universally employed in Norway than in most other countries, although the confidence in its efficacy as a preventive of small-pox has, during the last ten years, been diminished by the discovery that even those who have availed themselves of it may occasionally take that disease. The number vaccinated throughout the kingdom amounts to about 20,000 yearly. Revaccination, without being specially enjoined, has been frequently practised, especially during epidemic small-pox.

### HOSPITALS.

The National Hospital and the Lying-in Institution in Christiania have been already mentioned. The state contributes to them 4,450 specie-dollars yearly, as schools for the future medical practitioners and midwives of the kingdom. The National Hospital is unquestionably the most complete and best regulated in Norway. Each of the larger cities has one or more infirmaries for diseases in general; and almost every one of the seventeen districts has a distinct infirmary for syphilis, radesyge, and other malignant cutaneous diseases; but these are kept up at the charge of the respective cities or communes, and, generally speaking, stand in great need of essential improvements, with respect to situation, accommodation, and management.

Norway, like most other countries, is still very much behindhand in provisions for the cure of the insane. In consequence of a resolution of the Storthing, in 1824, his majesty, in the following year, nominated commissioners to enquire into the state of the lunatics throughout the kingdom, and propose measures for its improvement. The report of these commissioners, comprising general observations on the proper construction and management of lunatic asylums, the actual condition of the insane then throughout the kingdom, a proposal for its improvement, and a statement of the means whereby the requisite sums might be raised, was laid before the Storthing in 1827, but with no other result than its being ordered to be printed at the public charge; neither has there been anything done in the matter at any session since. However, there is reason to hope that the very publication of the Report has been of service, as it may be the occasion of awakening and diffusing profitable, and, to the majority of our countrymen, new ideas on a highly important subject, and of gradually making the nation better aware of the great necessity there is that something should be done.

There are at present in Norway seven lunatic asylums, which, with the exception of a new one established in Bergen in 1826, can scarcely be considered as anything but so many depôts for incurables.

By a census taken in 1826, the total number of lunatics in Norway was ascertained to amount to 1,909; that is, with respect to the actual population, one in every 551. Of the above number, 1,005 were males, and 904 females; and there were 512 stated as *maniaci*, 376 *melancholici*, 341 *dementes*, and 680 *idiotæ*.

### SCIENTIFIC SOCIETIES.

The Society of Sciences of Drontheim, (*Videnskabernes Selskab i Trondhjem,*) founded in 1760, and the Physiographical Association of Christiania, (*Den Physiographiske Forening i Christiania,*) in 1828, are interesting to the medical practitioner, not merely in a general literary point of view, but also because they embrace subjects relating to his own department; but the Medical Association of Christiania, (*Lægeforeningen i Christiania,*) founded in October, 1833, naturally presents a still stronger claim on his attention. This last meets regularly once a month, from September till May, and has besides extraordinary meetings not unfrequently. At these meetings papers are read and medical subjects discussed, and the Transactions are subsequently published. Christiania has also, since 1826, a Scientific Journal-Reading Society; and, since 1832, an Athenæum, which takes in medical works. It is worthy, too, of mention that, in 1829, the medical students at the university formed a Medical Improvement Society, the members of which assemble twice a month. This Society has a considerable resemblance to the Royal Medical Society of Edinburgh.

### VETERINARY ART.

As long as Norway and Denmark were united, those who wished to become veterinary surgeons were sent to the Royal Veterinary School in Copenhagen, for the purpose of receiving the requisite instruction. The very first year after the separation, in the session of the Storthing of 1815-16, a motion was made for a grant for the establishment of a similar institution in Norway; but other objects appeared to have stronger claims on the public purse, and accordingly Norway is yet without a veterinary school of her own. The consequence is, that pupils have still to be sent, as formerly, at the public charge, to Stockholm and Copenhagen, to learn the veterinary art, as practitioners in which they are subsequently established in Norway, after undergoing the requisite examination. In 1825, a young physician, *Candidatus medicinæ* C. B. Boeck, went, at the public expense, to Germany and France, for the purpose of spending two years there in the study of the veterinary art, in which he has been appointed lecturer since his return. At his suggestion, a shoeing forge was established at Christiania in 1832, at which instruction is given in the proper method of forming and putting on horse-shoes, as well as in the usual diseases to which horse's feet are subject. A collection has also been formed, under his superintendance, of zootomical preparations and veterinary instruments, in provision for the establishment of a school.

### MEDICAL ADMINISTRATION.

The administrative and executive medical department of Norway suffers in many respects from forms that are antiquated and inconsistent with the present state of the science; the oldest medical ordinance of the 4th of December, 1672, being still valid in most points. The necessity of a revision and reform of these regulations has, therefore, been for many years acknowledged. In 1834, a royal commission was appointed to draw up a proposal for a new and improved legislative enactment on the subject; and their report may perhaps be recommended for adoption to the Storthing of 1839. During the union, the medical concerns of Norway and Denmark were managed by the Royal Board of Health of Copenhagen, established in 1803. As the war that broke out in 1807 rendered the communication between the two countries tedious and uncertain, a Royal Board of Health was established in Christiania, in 1809. This was, however, dissolved again by the king, in 1815, by an edict which vested the management of these matters *pro tempore* in three official bodies; namely, the ecclesiastical and educational department, for civil affairs; the Army department, for military; and the Marine, for naval and such as relate to quarantine. At the same time, it was made incumbent on the faculty of medicine to assist the aforesaid bodies with such information and explanations as it might consider necessary; so that the faculty was appointed to the functions of medical assessor. Norway is, accordingly, unprovided with a board professionally acquainted with the subject to manage its medical concerns; a loss which has been constantly felt by persons of experience in such matters. I have even ventured myself to point out, in an essay written for the purpose, "The Necessity of the Establishment of a professionally informed Board of Health in Norway," (Eyr, 1833, pp. 324—352; also printed in a separate form;) and I feel confident that I have expressed in it the wishes and hopes of such of my countrymen as understand the subject.

Of medical practitioners who have civil employments, some are *Physici* (town or country), others district surgeons, others hospital surgeons. The number of these situations has considerably increased during the last twenty years; but almost all the present country physicianships and district surgeonships are much too extensive with respect to the space over which their charge extends, and should be divided into several less extensive ones, which it is hoped will gradually take place as the public finances may be found to admit of the requisite expense.

The army establishment consists of a surgeon-general, seven brigade surgeons, and a due proportion of corps and regimental surgeons; and the navy, of a chief surgeon, two corps surgeons, and some assistant surgeons. There are, besides, a class of private practitioners in Norway. According to the official lists, the number of licensed practitioners in the kingdom amounted, in 1816, to 99; in 1824, to 116; in 1833, to 129, (77 civil, and 52 military;) and at present may fairly be estimated at 140; a small number, certainly, (one to every 8,570 inhabitants,) in comparison with other countries, but which has yet increased at the rate of more than forty per cent. within the last twenty years.

The physicians and district surgeons receive from the public purse, or from their commune, a yearly salary of from 120 to 400 specie-dollars, according to the extent of their district and opportunities of private practice; the surgeon-general, 800 specie-dollars; the chief navy-surgeon, 600 *Spd.*; the corps-surgeons, 150 *Spd.*; the regimental surgeons, 180 *Spd.* The military medical officers receive, besides, lodging money, according to the nature of their appointments. For journeys on public business, dissections, and other legal examinations, there is a separate fee regulated by law. Every medical practitioner is likewise entitled to receive a certain honorarium from each individual whom he attends, which is usually detemined by agreement; but, when claimed by law proceedings, is estimated by rules laid down in the enactment of the 4th of December, 1672.

Under the immediate control of the medical practitioners are the apothecaries' shops, of which there are at present thirty-seven licensed in Norway, all in towns. In the country, and in towns where there are no such shops established, the practitioners themselves have to dispense medicines to their patients.

In the preparation of medicines, the *Pharmacopœia Danica* (Hafniæ, 1805,) is still followed; but, as it is no longer adapted to the present state of medical knowledge, a royal commission was established some years ago to compose a *Pharmacopœia Norvegica,* which is now so far advanced that there is reason to hope it will be soon finished. Medicines are dispensed by the apothecaries only when prescribed by authorized medical practitioners, with the exception of a few simple ones specially named in the enactment. They are bound by law to charge for them according to a scale of prices, constructed after certain rules by the ecclesiastical and educational department, and altered as often as a fall or rise in the price of drugs renders it necessary.

It is incumbent on all the licensed practitioners in the kingdom, whether in official stations or in private practice, to send in yearly, to the Ecclesiastical and Educational Department, a medical report containing as full and accurate information as their opportunities afford respecting the epidemic, endemic, and contagious diseases of the year, as well as the state of the weather, the quality of provisions, and manner of living; also respecting the attendance on the poor, management of disease, mortality, bathing regulations, vaccination, and everything relating to apothecaries, midwives, examinations of dead bodies and other medico-forensic proceedings, quackery, &c. From these the department compiles a General Report, which is laid before the king, and afterwards printed in the Gazette of that department and in the "Eyr."

LITERATURE.

The medical literature of Norway is but of small extent, and will probably always continue such in proportion to the population; as the latter is not great,* the medical practitioners are but few, and the language of the country is understood by very few out of Scandinavia. Consequently, neither the medical man who might feel inclined to come forward as an author, nor the bookseller who is able to undertake the cost of printing the work, has the same encouragement as in other countries. It would, therefore, be unjust to consider the paucity of literary productions in that country as a proof of the incapacity of its medical men. The facilities of communication with other countries makes them pretty soon acquainted with foreign publications on subjects in their department; and we have no well-informed practitioner who does not take one or more of the best foreign Medical Journals, and procure the more important works by foreign medical writers. The medical works that have appeared in Norway have, in almost every instance, been called forth by particular occasions, or possess merely a private or local interest. The following are those that have been published since 1814.

J. G. Doederlein: De Catarrhi theoria. Diss. pro licentia.—Christianiæ, 1816. 8vo. Chr. Heiberg: 1. De Coremorphosi. P. i. Diss. pro licentia.—Christianiæ, 1827. 8vo. 2. De Coremorphosi. P. ii. Diss. pro gradu Doctoris.—Christianiæ, 1829. 8vo.

J. J. Hjort: 1. De Functione Retinæ. P. i. Diss. pro licentia.—Christianiæ, 1826. 8vo. 2. De Functione Retinæ. P. ii. Diss. pro gradu Doctoris.—Christianiæ, 1830. 8vo.

Fredericus Holst: 1. De Acidi Nitrici usu Medico. Diss. pro licentia.—Christianiæ, 1816. 8vo. 2. Morbus, quem Radesyge vocant, quinam sit, quanamque ratione e Scandinavia tollendus? Diss. pro gradu Doctoris.—Christianiæ, 1817. 8vo. 3. Remarks on the Modern Prisons of Great Britain, with especial Reference to the Necessity of an Improvement in Prison Discipline in Norway; with two lithographic Plates.—Christiania, 1823. 8vo. xviii. 234.

4. Historical Account of the National Hospital at Christiania.—1827. 8vo. xii. 148; with a lithographed Plate.

5. Report of the Commission appointed by the King, in 1825, to examine the Condition of Lunatics in Norway, and propose a Plan for its Improvement. With

* A general census has as yet been taken but at five periods in Norway, namely:

| | |
|---|---|
| The 15th of August, 1769, when the population was found to amount to | 733,141 |
| The 1st of February, 1801, | 883,038 |
| The 30th of April, 1815, | 885,431 |
| The 27th of November, 1825, | 1051,318 |
| The 29th of November, 1834, | 1194,812 |

The area of the country is equal to 5,742 square geographical miles, and consequently there are but 208 inhabitants to each square mile.

a Collection of Tables, and two lithographed Plates. Published by his Majesty's Orders.—Christiania, 1828. 8vo. xiv. 139.

N. B. Sörenssen: Collegium Academicum Universitatis Regiæ Fredericianæ, summos in Medicina honores Licentiato Frederico Holst, die xviii. Junii, 1817, conferendos indicat.—Christianiæ, 1817. 4to. A Program, in which the author treats of an affection of the neck often wrongly considered as syphilitic.

Provisional Regulations for the National Hospital at Christiania.—Christiania, 1826. 8vo. 66.

S. F. Hanstein: The Indian Cholera; a Work for the People.—Drammen, 1832. 4to.

*Magazin for Naturvidenskaberne*, (The Magazine for Natural History.) This publication, which was commenced in 1823, was at first a quarterly one, but now comes out at uncertain periods. It was originally edited by Professors Hansteen, Lundh, and Maschmann, and afterwards by Lecturer Boeck, but has been latterly conducted by the Physiographical Association.

*Eyr*, a Medical Gazette, commenced in 1826, and for the first five years edited by Professors Skjelderup and Holst, but afterwards by Professor Holst alone. During the first ten years, (that is, up to 1835 inclusively,) the numbers came out quarterly; but they are henceforth to be published at irregular periods, just as sufficient materials are procured, and to consist principally of original articles by Norwegian authors, and information concerning the medical affairs of the country.

*Budstikken*, (The Express,) a weekly paper on statistical, economical, and historical subjects; it also contains various medical articles.

This short account of the state of medical affairs in Norway shows that there is room for improvement in many respects. At the same time, however, it must be allowed that, on the whole, considerable progress has been made during the last twenty years. Thus, the Faculty of Medicine, by the increase in the number of its professors, has been enabled to deliver instruction, both in a more complete manner and in a shorter time; more medical situations have been established, and places, before destitute of apothecaries' shops and midwives, provided with the same; while the nation at large has become possessed of a much greater number of skilful physicians, apothecaries, and midwives, as well as some better organized infirmaries, &c.

*Christiania; October*, 1836. F. H.

[The present Report was forwarded from Christiania last October, but the vessel which conveyed it was forced back when near the English coast, and obliged to winter in Norway: consequently, the Report did not reach us until the beginning of the present summer. For the foregoing translation of this interesting document from the original Danish, we are indebted to our most learned and excellent friend, Dr. West, of Dublin, whose extensive acquaintance with modern languages is well known.—Ed.]

---

